# Formation of free-floating planetary mass objects via circumstellar disk encounters

**DOI:** 10.1126/sciadv.adu6058

**Published:** 2025-02-26

**Authors:** Zhihao Fu, Hongping Deng, Douglas N. C. Lin, Lucio Mayer

**Affiliations:** ^1^ Department of Physics, The University of Hong Kong, Hong Kong, China.; ^2^ Shanghai Astronomical Observatory, Chinese Academy of Sciences, Shanghai 200030, China.; ^3^ Department of Astronomy and Astrophysics, University of California, Santa Cruz, Santa Cruz, CA 95064, USA.; ^4^ Institute for Advanced Study, Tsinghua University, Beijing 100084, China.; ^5^ Department of Astrophysics, University of Zurich, Zurich 8057, Switzerland.

## Abstract

The origin of planetary mass objects (PMOs) wandering in young star clusters remains enigmatic, especially when they come in pairs. They could represent the lowest-mass object formed via molecular cloud collapse or high-mass planets ejected from their host stars. However, neither theory fully accounts for their abundance and multiplicity. Here, we show via hydrodynamic simulations that free-floating PMOs have a unique formation channel via the fragmentation of tidal bridges between encountering circumstellar disks. This process can be highly productive in dense clusters like Trapezium forming metal-poor PMOs with disks. Free-floating multiple PMOs also naturally emerge when neighboring PMOs are caught by their mutual gravity. PMOs may thus form a distinct population that is fundamentally different from stars and planets.

## Teaser

Free-floating planetary mass objects have their own formation channel, neither star-like nor planet-like.

## INTRODUCTION

Free-floating planetary mass objects (PMOs) with masses below the deuterium burning limit [13 Jupiter mass (*M*_J_)] are discovered via both direct imaging (*1*, *2*) and microlensing (*3*, *4*). Isolated PMOs that are more massive than Jupiter, lying at the border between stars and planets, are particularly interesting. Such PMOs glowing in infrared were frequently observed in nearby young star clusters by the James Webb Space Telescope (JWST) (*5*–*8*). Are they representatives of the low-mass end of the star formation process or unlucky giant planets exiled by their parent stars?

Stars formed by molecular cloud collapse follow characteristic mass functions. However, in the 10 to 20 *M*_J_ mass range, isolated objects can be excessively abundant, as observed in the Trapezium cluster (*9*), and free-floating PMOs in the Upper Scorpius stellar association are up to seven times more abundant than the mass functions’ prediction (*2*). As a result, an extra formation channel is required to explain the rich PMO population. In addition, free-floating multiple PMOs and candidates were reported with projected separations ranging from several to hundreds of astronomical units (au) (*5*, *7*, *10*–*12*). The high multiplicity (~9%) of PMOs in the Trapezium cluster, though needs further confirmation, defies a star-like origin since stellar multiplicity decays with stellar mass, and the extrapolation wide binary fraction for PMOs should be close to zero (*13*).

Nor are they mature planets ejected after dynamic interactions. PMOs formed as ejected planets would have spatial and kinematic distributions distinct to those of stars, contrasting with the PMOs’ distribution in NGC 1333 (*14*). Dynamic simulations of the Trapezium cluster also suggest the concurrent formation of PMOs with stars (*15*), further supported by the prevalence of extended gaseous disks around PMOs (*16*–*18*). To explain free-floating PMOs via planet ejection begs knowledge of the population of wide-orbit giant planets in the first place, which is poorly constrained. Nevertheless, to explain the PMO population in the Trapezium cluster, an unrealistically high occurrence rate of wide-orbit (>100 au) giant planets is required (*19*, *20*) in contrast with observations (*4*, *21*).

## RESULTS

**Fig. 1.**
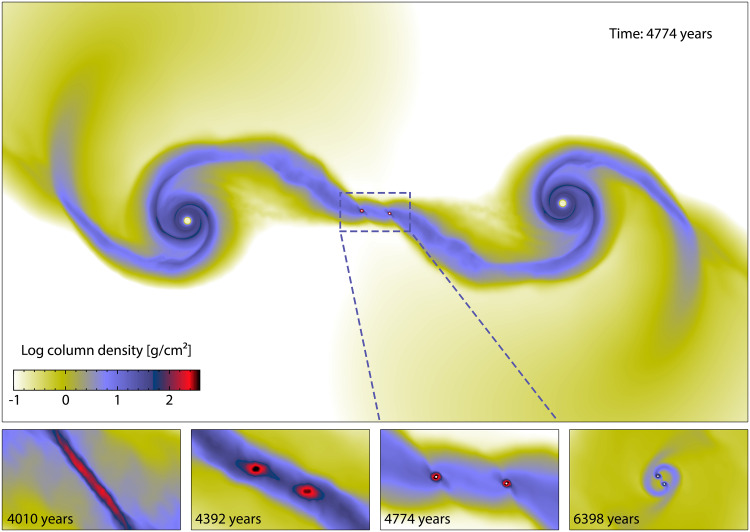
The formation of binary PMOs via circumstellar disk encounters. The upper panel shows the density map (2000 au × 1120 au) in a logarithmic scale for an example of a simulation (model rp400v2.65 of table S1). The lower panels enlarge a region of 200 au × 112 au to show the evolution of the binary PMOs, sink particles in white with exaggerated radii, and how they emerge within the dense filament created by the encounter (see movie S1).

Here, we propose a scaled-down version of filament fragmentation, the modern theory of star formation (*22*), as a possible means to form free-floating PMOs, including multiples. When the mass per unit length (line mass) of filamentary molecular clouds is beyond a critical value, $M_{\text{crit}}=\frac{2c_{s}^{2}}{G}\;$ (here, *c*_s_ is the sound speed and *G* is the gravitational constant), they fragment on a scale about four times the filament diameter to form dense cores and eventually stars (*23*). The typical width of such a filament is 0.1 parsec (pc) with a column density of 0.001 g/cm^2^ (*24*). To fragment into PMOs, a filament 0.001 times thinner (20 au) with a column density around 1000 g/cm^2^ is necessary, assuming a comparable temperature. We show with hydrodynamic simulations that such filaments are naturally produced in the tidal bridge connecting two encountering young circumstellar disks. The filament further fragments into PMOs, sometimes forming binary and even triple PMOs (Fig. 1).

### From disk encounters to dense filaments and cores

Many young circumstellar disks are prone to instabilities due to the self-gravity of disk gas (*25*, *26*), potentially leading to disk fragmentation and the formation of gaseous planets (*27*, *28*). Circumstellar disks appear even more unstable when perturbed by a stellar or circumstellar disk flyby. These flybys can induce the formation of PMOs in disks that are otherwise stable in isolation (*29*–*32*). However, brown dwarfs instead of PMOs are often formed in these early studies, and PMOs are only marginally resolved if they are not spurious fragments due to poor resolutions (*32*–*34*).

**Fig. 2.**
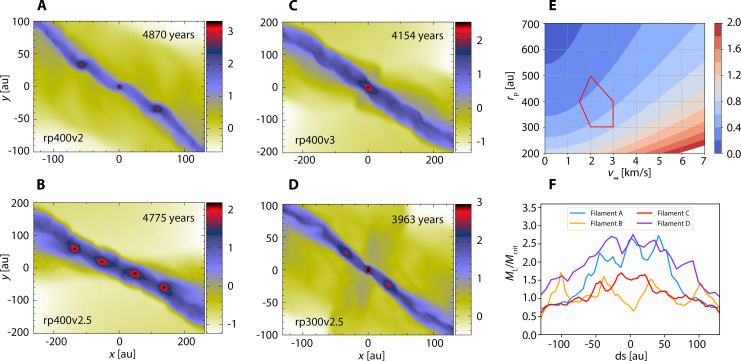
The formation of compact cores within dense filaments under different encounter conditions. (**A** to **D**) show the central region gas column density in g/cm^2^ in a logarithmic scale for representative simulations in table S1. They have a series of dense cores comparable to the Fig. 1 insert panel at 4392 years. (**E**) The encounter parameter space that forms FFOs via filament collapse within red curves (table S1); the background contours show the peak angular speed of different flybys divided by the Keplerian frequency at 100 au. (**F**) Line mass of precursors of the (A) to (D) filaments (~400 years earlier; see also Fig. 1) normalized to the critical line mass for stability (see main text).

We performed a series of hydrodynamic simulations of circumstellar disk encounters with the meshless finite mass (MFM) scheme (*35*) at a mass resolution of 0.0001 Jupiter mass, improved by over an order of magnitude compared to the previous studies. In addition, we focus on the extended tidal bridge, which forms most effectively in near coplanar prograde encounters when the disk’s body is in quasi-resonance with the flyby orbital motion (*36*, *37*). Informed by observations of the Orion Nebula Cluster (ONC), we construct models of disks that are marginally stable in 100 to 200 au (fig. S1) and surround low-mass stars of ~0.3 solar mass (*38*, *39*). These disks are set onto hyperbolic encounter orbits with periapsis distance *r*_p_ = 200 to 500 au and velocities at infinity *v*_∞_ = 1 to 5 km/s (table S1 and Fig. 2).

Previous studies show that Keplerian motion is most strongly perturbed when the peak angular velocity of the flyby is about 0.6 times the Keplerian frequency (*37*). For encounters with *v*_∞_ = 2 to 3 km/s and *r*_p_ = 300 to 400 au, the peak orbital angular speed (at periapsis) is right about 0.6 times the disk Keplerian frequency at 100 au, causing strong tidal perturbations (Fig. 2E). Besides the periapsis, the orbital angular speed is lower than the peak value (fig. S2), so the quasi-resonance will sweep the whole region beyond 100 au. As a result, the disks each form two grand spiral arms, and the neighboring arms join to form an extended tidal bridge (Fig. 1 and movie S1).

The middle part of the tidal bridge contracts into thin filaments with line mass over the critical value for stability (Fig. 2F), forming up to four cores in one encounter (Fig. 2). However, the exact number of compact cores is determined by the length of the filaments and is sensitive to random density fluctuations (*23*), which is barely predictable from encounter parameters. For example, varying *v*_∞_ by only 50 m/s in the rp400v2.65 model can lead to different results of one core, two cores, and no cores, as shown in table S1.

For high- and low-velocity encounters, the tidal bridge is either stretched too thin or torn apart by the stars, and thus, forming isolated cores becomes impossible (fig. S3). The Trapezium cluster with an observed velocity dispersion of 2 to 3 km/s (*40*, *41*) hits the sweet spot for forming isolated cores via disk encounters (Fig. 2E).

### PMO properties

**Fig. 3.**
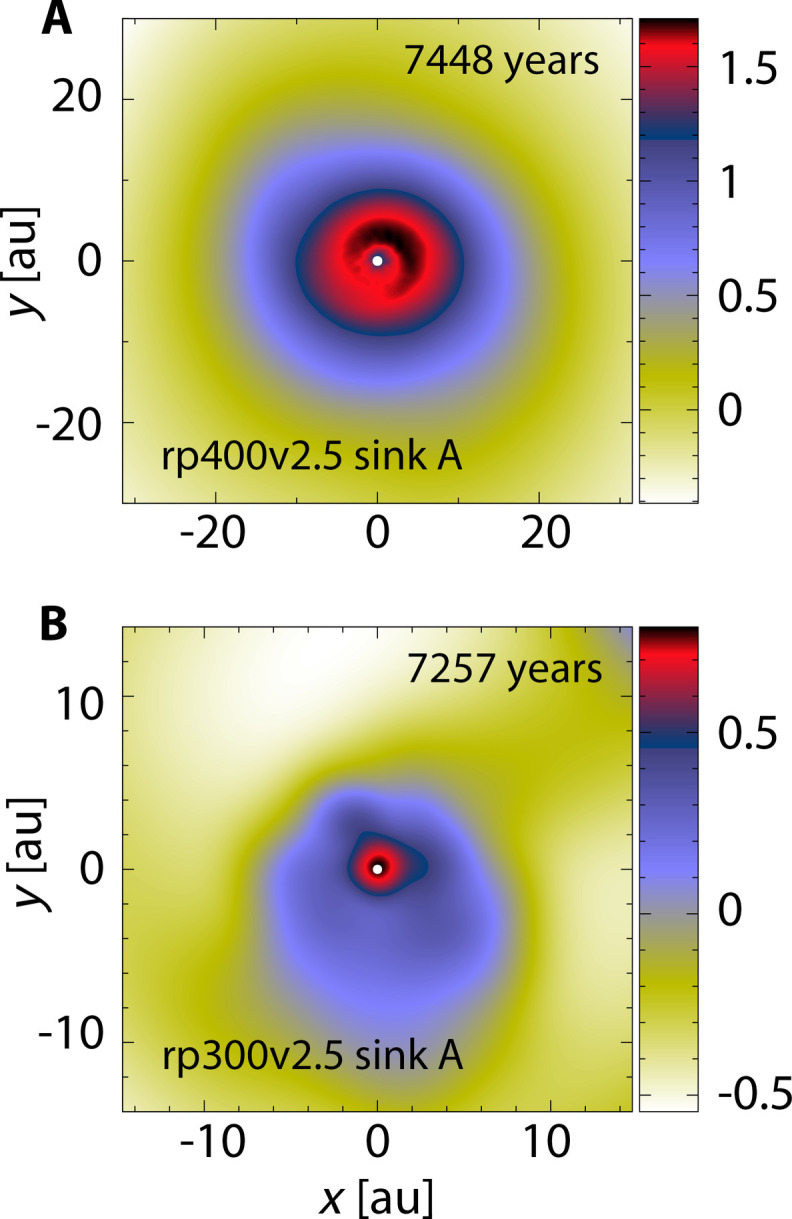
Disks around free-floating PMOs. (**A** and **B**) Similar column density maps like Fig. 1, centered on free-floating PMOs at the end of two simulations (table S1). The PMOs can have extended disks, and the PMO disk in (B) is lopsided because of perturbations from the other PMO (to the upper right, not shown here) in this binary system (see another binary in Fig. 1).

The dense cores collapse spherically in almost all simulations within ~400 years (Fig. 1), leading to prohibitively small simulation time steps in the core center. To follow the motion of isolated cores further, we have to introduce sink particles (fig. S4) at the center of dense cores (*42*, *43*). We follow the system until the parent stars are well separated by >2000 au and then determine the free-floating objects’ (FFOs) mass (likely overestimated; figs. S4 and S5), boundness, and disk property (Fig. 3).

As shown in Fig. 2, the coplanar prograde encounters are very productive in forming isolated dense cores. For *r*_p_ = 300 to 400 au, *v*_∞_ = 2 to 3 km/s, almost every encounter (see table S1) produces an FFO. These FFOs have slow velocities relative to the parent stars and can naturally blend into the cluster (*14*). PMOs preferentially form in filaments with at least two cores featuring competing accretion; otherwise, low-mass brown dwarfs of ~20 *M*_J_ are formed. This result aligns with the overabundance of FFOs of mass 10 to 20 *M*_J_ seen in the Upper Scorpius stellar association (*2*) and Trapezium cluster (*9*). On the other hand, nonsymmetric encounters involving two different disks tend to form a higher fraction of PMOs than the fiducial symmetric encounters (table S2 and fig. S5). However, the exact masses of FFOs formed via disk encounters can be sensitive to gas thermodynamics, determining the filament’s width and fragmentation scale (fig. S6). PMOs are exclusively produced at high efficiency (up to seven free-floating PMOs per encounter) in test simulations with local isothermal gas (table S3). Other physical mechanisms to limit the mass of FFOs to the planetary mass regime may include magnetic fields, which appear efficient in a similar process of disk spiral arm fragmentation (*28*).

Our fiducial simulations formed four binaries among the 33 FFOs (table S1), and their semi-major axes of their orbits are 7 to 15 au (table S4). The multiplicity fraction is 13.8 ± 6.9% (one-sigma uncertainty), comparable to the inferred multiplicity of 9% in the 1 million years (Myr) old Trapezium cluster. The multiplicity fraction is even higher if we focus on PMOs, which preferentially form in a chain of competing cores (Fig. 2). Their separations may expand to hundreds of astronomical units like those in the Trapezium cluster, and a fraction of them become ionized after interacting with cluster stars for millions of years (*44*). On the other hand, the chaotic isothermal simulations, featuring interactions among a chain of close-packed PMOs, preferentially form multiple PMOs, including hierarchical triple and quadruple systems (fig. S7 and table S4). The tight binaries in the hierarchical triple PMOs have semi-major axes <4 au (*10*), while the tertiary PMO is loosely bound and may leave the system (*44*).

Extended disks form around PMOs in our fiducial simulations (Fig. 3 and table S1), with low-density gas filling the PMOs’ Hill radii up to 200 au (*16*). These disks have a characteristic surface density profile, which is nearly flat within 10 au and then declines quickly, following a *R*^−2^ scaling (fig. S8); the disks show Keplerian rotation within 10 au and then transit to sub-Keplerian rotation. On the other hand, disks around multiple PMOs are dynamic and eject mass into the ambient environment at every periapsis crossing of PMOs (movie S1). In addition, PMOs and their hosts are expected to be metal-poor since they inherit materials in the parent disks’ outskirts that are susceptible to dust drift and, thus, are metal-depleted (*45*). The disk may quickly lose most of its mass when it is subject to photoevaporation, as in the Trapezium cluster (*16*), and potentially leave traces as tiny molecular clouds, i.e., globulettes (*46*). As a result, it is uncertain if they can form scaled-down planetary systems in the long term. Nevertheless, the characteristic disk size, steep density profile, and metal-poor nature confronted with observations (*16*–*18*) may help to tell if PMOs are formed via disk encounters.

We further tested the effect of misaligned disks to assess PMO formation efficiency in general encounters. Slightly misaligned encounters evolve similarly to coplanar encounters, as shown in Fig. 1, but form less dense tidal bridges. When the mutual inclination of disks is smaller than the disk opening angle (about 5° here), FFOs can form via tidal bridge collapse (table S5). However, this should not be regarded as a solid criterion since encounters with more massive disks can form more critical filaments and still fragment into FFOs.

## DISCUSSION

In the Trapezium cluster, we can estimate the close encounter rate for *r*_p_ < 500 au, as σ_v_*n*π*b*^2^; here, we take a stellar density *n* = *5* × 10^4^ pc^−3^, velocity dispersion σ_v_ = 2.5 km/s (*40*), and *b* is the corresponding impact parameter. We find that every star experiences 3.6 encounters on average within 1 Myr, i.e., the lifetime of Trapezium. We estimate ~10% of the encounters to be nearly coplanar (relative angle <5°), informed by the distribution of relative angles for binary disks in star cluster simulations (see Supplementary Text) (*47*). The highly efficient PMO production channel via encounters (Fig. 2 and fig. S6) can therefore explain the hundreds of PMO candidates (540 over 3500 stars) observed in the Trapezium cluster. In addition, PMOs so formed have a high initial multiplicity fraction that can account for the present-day PMO binary fraction in Trapezium even after some ionization.

The Upper Scorpius Association has the next largest known population of free-floating PMOs, second to the Trapezium cluster. It also features a velocity dispersion (*48*) amenable for PMO formation via disk encounters (Fig. 2). Nevertheless, the IC 348 and NGC 1333 clusters have smaller velocity dispersions <1 km/s (*49*, *50*), affecting the encounter rates and PMO formation efficiency, and leading to a smaller population of PMOs than that in Trapezium. This outcome is particularly true for IC 348, which has a low stellar density. Future studies of various young clusters (*41*) can further constrain the population of PMOs.

## MATERIALS AND METHODS

### Numerical methods

We used a Godunov-type Lagrangian method, the MFM scheme (*35*), to solve the self-gravitating hydrodynamics in two interacting massive circumstellar disks. Specifically, the simulations are performed with the public hydrodynamic code GIZMO, where gas self-gravity is efficiently calculated via a Tree algorithm with adaptive gravitational softening (*35*, *51*, *52*). The MFM method has been successfully deployed in modeling star cluster formation (*43*) and gravitationally unstable circumstellar disks focusing on disk fragmentation (*28*, *53*). Notably, MFM can avoid artificial fragmentation even in poor resolution, producing consistent results across a wide range of resolutions (*43*, *53*). In contrast, artificial fragmentation often occurs in low-resolution smoothed-particle hydrodynamics (SPH) simulations (*32*–*34*). Because of MFM’s Lagrangian nature and good conservation property, we managed to cover a size range of 0.1 au (in disks around PMOs; Fig. 3) to >2000 au in these encounter simulations (Fig. 1).

For simplicity, an idealized barotropic gas equation of state (EOS) (*54*), calibrated to radiation-hydrodynamic simulations of the collapse of a protostellar core of 1 solar mass (*55*), is used to allow an extensive suite of high-resolution simulations. However, this EOS likely underestimates the cooling efficiency (*56*) because (i) the PMO cores are much less massive than protostellar cores, and (ii) both the cores and the tidal bridge hosting them should be metal-poor and thus have low opacity (*45*). Thus, we also provide isothermal simulations for comparison. We note that the collapse of isothermal filaments cannot be halted by the gas pressure (*23*), so the filament width keeps decreasing until small-scale supersonic turbulence provides support at a certain point. Thus, the filament width and fragmentation scale in the isothermal simulations are quite uncertain. More detailed radiative hydrodynamic simulations are desirable in the future and are expected to give results between our barotropic and isothermal simulations.

As the dense cores collapse (Fig. 1), the time steps within the cores become very small, preventing long-term simulations, so sink particles are introduced at their centers per the criteria of (*43*). We use a simple accretion criterion (*42*) for all sink particles accreting material within a sink radius of 0.5 au (10 au for the stars hosting the disks). The mass and momentum of accreted materials are added to the sink particle to ensure mass and momentum conservation. This approach inevitably exaggerates the accretion of the sink particles. As a result, their masses should be regarded as the upper limits of the objects’ actual masses.

### Initial conditions

We use observationally informed parameters for the circumstellar disks, which closely represent conditions in the Trapezium cluster, where many PMOs have been observed. We consider young circumstellar disks given the young age of the Trapezium cluster of about 1 Myr. They are relatively massive and likely hover around the edge of gravitational instability (Supplementary Text) (*26*, *57*, *58*). The disks are assumed to follow a self-similar surface density profile (*59*).

$$\Sigma \left(R\right)=\Sigma_{0}{\left(\frac{R}{R_{c}}\right)}^{-\gamma }\text{exp}\left[-{\left(\frac{R}{R_{c}}\right)}^{2-\gamma }\right]$$

Here, we set γ = 0.9, *R*_c_ = 200 au, and the disk extends from 20 to 600 au (*58*, *60*). The disk mass is 0.18 (0.2) solar mass surrounding a central star of 0.3 (0.33) solar mass (*39*). They are named by the host mass and disk mass as m_s_0.3m_d_0.18 or m_s_0.33m_d_0.2 (fig. S1).

Each disk is represented by 1.8 million or 2 million fluid particles required to resolve the disk’s vertical structure and avoid spurious fragmentation (*32*–*34*). Equivalently, a 10 Jupiter mass PMO is resolved by 100,000 particles. We also considered low-resolution models with particles two times more massive. We realized the disk density structure via rejection sampling following (*34*). To reduce the particle noise, we relaxed the initial condition for 5000 years by damping the vertical and radial motion at every time step to reach an equilibrium state.

These disks are close to gravitational instability with an initial Toomre Q parameter (*61*) of about 2 (fig. S1) between 100 and 200 au (*25*, *26*). We also considered for tests an isothermal disk with a temperature profile proportional to *R*^−0.6^, where the temperature at 1 au is assumed to be 300 K and a minimum temperature of 10 K is applied at the outer part (*34*). Over 90% of the disk mass resides within 400 au, about the upper limit of the observed disk size in the Trapezium cluster (*38*). However, we note that the encounters themselves will notably truncate the disks, as shown in fig. S2. In addition, long-term photoevaporation likely shrinks the disks in size further (*62*), so we believe that the adopted disk size is reasonable.

### Simulation setup and analysis

We place isolated circumstellar disks on prescribed hyperbolic trajectories with an initial separation of 2000 au. Here, we focus on coplanar encounters with prograde spins for both disks, often leading to long tidal bridges critical for PMO formation. However, previous studies focused on noncoplanar cases (*31*) or encounters with retrograde disk spin (*32*).

These hyperbolic orbits are characterized by their periapsis distance (*r*_p_) and velocity at infinity (*v*_∞_). Our fiducial simulations cover *r*_p_ ~ 200 to 500 au and *v*_∞_ ~ 1 to 5 km/s for encounters involving two m_s_0.3m_d_0.18 disk models (fig. S1), and we summarize the results in table S1. These simulations illuminate the critical physics of PMO formation and identify the relevant parameter space. We then studied encounters involving two different barotropic disks (see fig. S1) but focused on *r*_p_ ~ 300 to 400 au and *v*_∞_ ~ 2 to 3 km/s, i.e., the PMO productive region of table S1. These nonsymmetric encounters are summarized in table S2. We further tested the effect of gas thermodynamics by using the isothermal disk model in fig. S1 and present the results in table S3. Finally, we explored a few cases of noncoplanar encounters in table S5 and performed simulations at a lower resolution (using ~2 million particles) to study the resolution effect in table S6.

We simulate the encounter until the stars are separated by >2000 au again or up to 7500 years (only for low-velocity encounters unable to produce FFOs). The duration of the simulation is primarily limited by the computational cost. Each encounter simulation takes up about 20,000 central processing unit (CPU) hours on the ShanHe supercomputer of the Chinese National Supercomputing Center, Jinan. The stars follow the hyperbolic trajectory well before the encounter (fig. S2), while the orbit tightens due to later tidal interactions (*63*). After the encounter, *v*_∞_ typically declines by 0.1 to 0.3 km/s for encounters with *r*_p_ ≥ 300 au and can decline by 0.6 km/s for a close encounter with *r*_p_ = 200 au. Near the end of the simulations, the tidal bridge is substantially dispersed with a column density below 1 g/cm^2^ (Fig. 1). We then check if the formed objects are unbound to the parent stars to become FFOs. We also check if FFOs are bound to each other to form multiples. Disk masses for single FFOs are calculated by weighting gas within its Hill radius with respect to the nearest star.

## Supplementary Materials

### The PDF file includes:

Supplementary Text Figs. S1 to S8 Tables S1 to S6 Legends for movies S1 and S2 References
