# Supplementary material for: Formation of free-floating planetary mass objects via circumstellar disk encounters
Source: Sci Adv. 2025 Feb 26;11(9):eadu6058. doi: 10.1126/sciadv.adu6058 (PMC11864182; doi:10.1126/sciadv.adu6058)
Supplement: Supplementary file 1 — Supplementary Text Figs. S1 to S8 Tables S1 to S6 Legends for movies S1 and S2 References [file sciadv.adu6058_sm.pdf]

Science Advances
AAAS

Supplementary Materials for

Formation of free-floating planetary mass objects via circumstellar disk encounters

Zhihao Fu *et al.*

Corresponding author: Hongping Deng, hpdeng353@shao.ac.cn

Sci. Adv. **11**, eadu6058 (2025)
DOI: 10.1126/sciadv.adu6058

The PDF file includes:

Supplementary Text
Figs. S1 to S8
Tables S1 to S6
Legends for movies S1 and S2
References

Other Supplementary Material for this manuscript includes the following:

Movies S1 and S2

Supplementary Text

Estimations for the probability of near coplanar encounters

The statistics of the mutual inclination between encountering disks are poorly constrained by both observations and theory. However, the pairs of disks encountering each other should have spins correlated instead of randomly drawn. The reason is twofold: 1) close-by stars/disks forming in the same molecular cloud filaments have a higher probability of encountering their close siblings than a random star farther away (assuming a stellar velocity dispersion of 2 km/s like in the Trapezium cluster, the distance a star can travel in 1 myr is only 2 pc comparing to the cluster size of ~400 pc); 2) stars/disks forming in the same molecular cloud filaments mostly have spin perpendicular to the initial filament axis (*64*), i.e., they have almost aligned spins.

Although the distribution of mutual angle between encountering disks is unknown, the mutual angle between binary disks can be a reasonable approximation because recent observations show that the relative angle between binary disks is independent of the projected separation (*65*). Notably, the orientation of binary disks is correlated instead of randomly drawn (*66*); otherwise, we would not expect the two near coplanar binary disks among the seven binary disks of Ref *65*.

To roughly estimate the probability of near coplanar encounters, we turn to the star cluster formation simulation by Ref *67*. We utilize the distribution of relative angles between binary disks (Fig. 19 of Ref *47*) to estimate a probability of near coplanar encounters of ~10%. The PMO production rate by encounters can vary due to disk property, thermodynamics, and encounter geometry (table S1-S3, table S5). In general, a coplanar encounter probability of a few percent agrees with the PMO fraction in Trapezium.

On the lifetime of marginally gravitationally stable disks

Our disk models are marginally gravitationally stable (fig. S1), which is the likely state of early disks subject to infall maintaining a balance between material loading and transport (*26,68*). By early disks, we refer to class 0 and class I disks, covering the first 1 myr evolution. For example, the class I HL tau disk is fed by infall and streamers (*69*) and is still close to gravitational instability (*70*). These young disks are massive with disk-to-star mass ratios > 0.1 (*70,71*).

On a population level, recent studies suggest that some disks of several myr old may still be massive enough and are at the cusp of gravitational instability (*58*). As a prominent example, the 2.5–4.4-myr-old AB Aurigae can have a disk of up to a third of the stellar mass (*57*). In summary, Class II marginally gravitationally stable disks are probably no more than 10-20% of the population, but Class I objects are susceptible to gravitational instability for a few hundred thousand years (*68*), and streamers may extend that state beyond 1 myr. As a result, it is safe to assume that the encounters that happened within the 1 myr old Trapezium cluster involve disks marginally gravitationally stable.

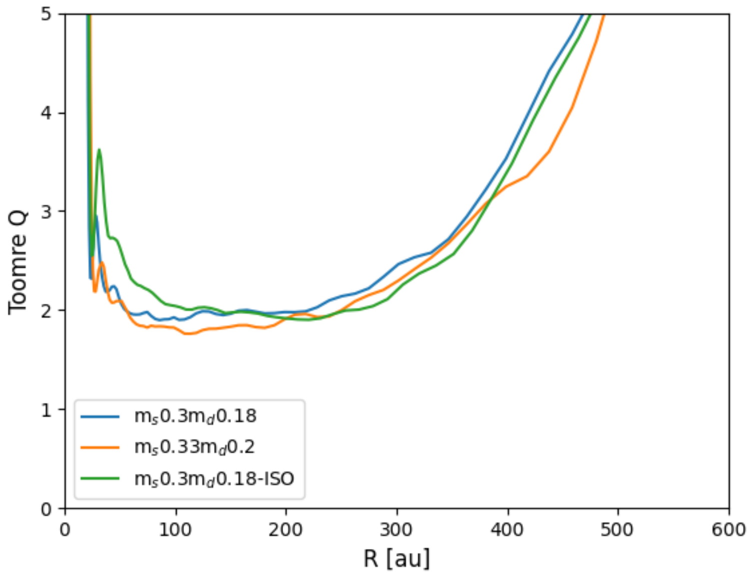

Fig. S1.

The Toomre Q profiles for isolated disk models with different mass and EOS. The disks are marginally gravitationally stable in the 100-200 au region. The legend indicates the host mass (m_s) and the disk mass (m_d), and the suffix "ISO" indicates an isothermal model.

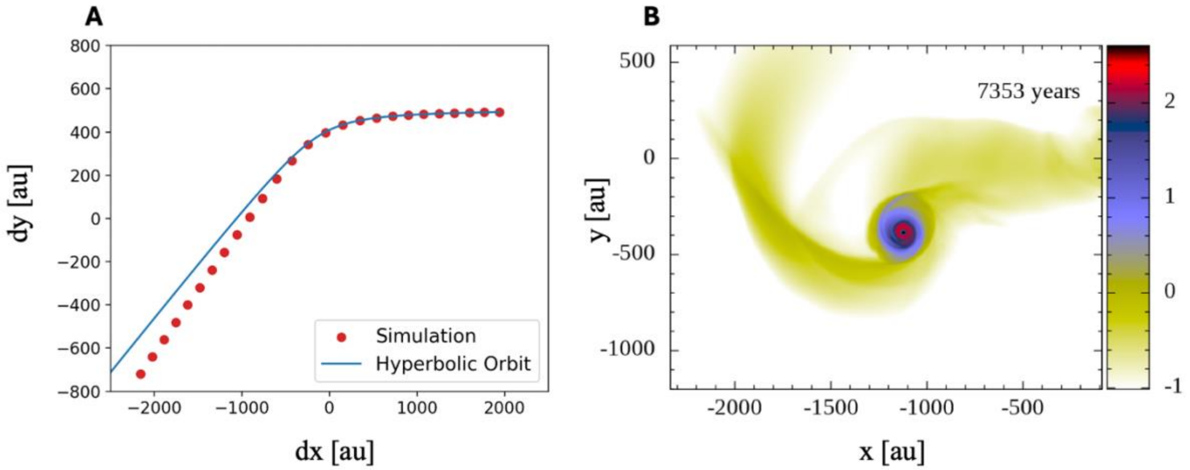

Fig. S2.

The relative trajectory of the stars (A) and the disk morphology at the end of the Fig. 1 simulation (B). The time interval between two consecutive simulation snapshots in (A) is 286 years. The post-encounter disk possesses two streams of material extending beyond 1000 au which should be distinguished from infall.

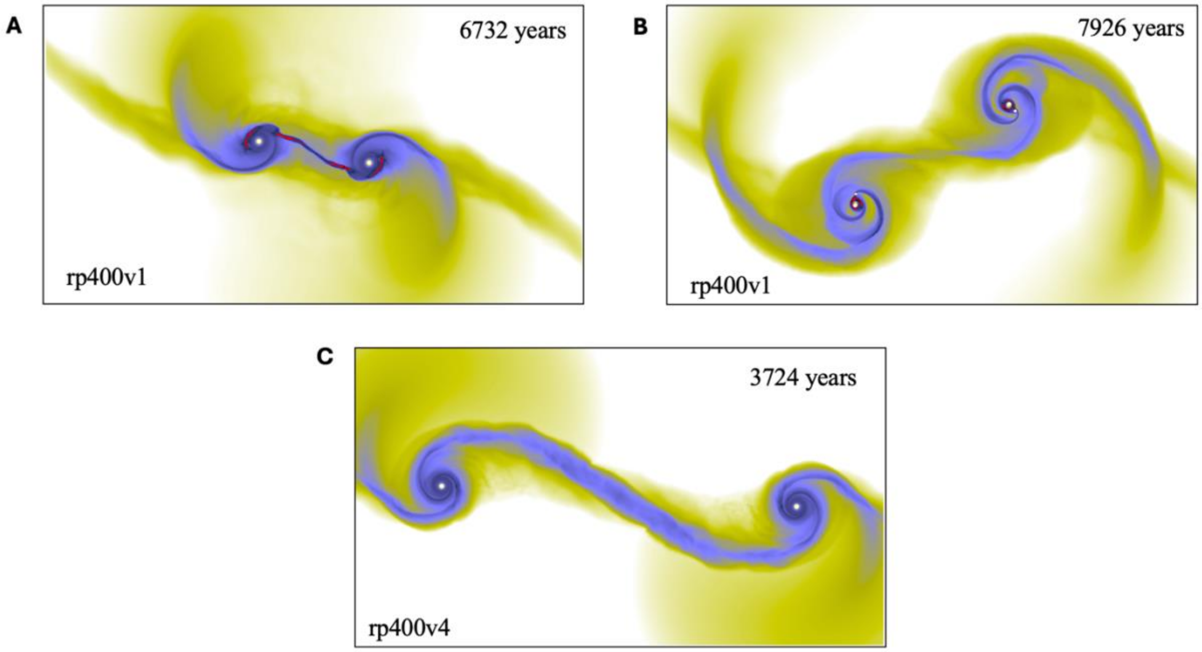

Fig. S3.

The formation of bound companions in a slow encounter (A, B) and the diffusive tidal bridge in a fast encounter (C). Each panel shows the logarithm of the surface density in g/cm^2 covering a region of 2000 au × 1120 au centered on the origin (like Fig. 1). In the low-speed encounter with $v_\infty = 1$ km/s, the tidal bridge is short. It is torn apart by the nearby star and eventually collides with the other spiral arms to form bound companions. However, the long tidal bridge in fast encounters is quickly dispersed due to fast stretching.

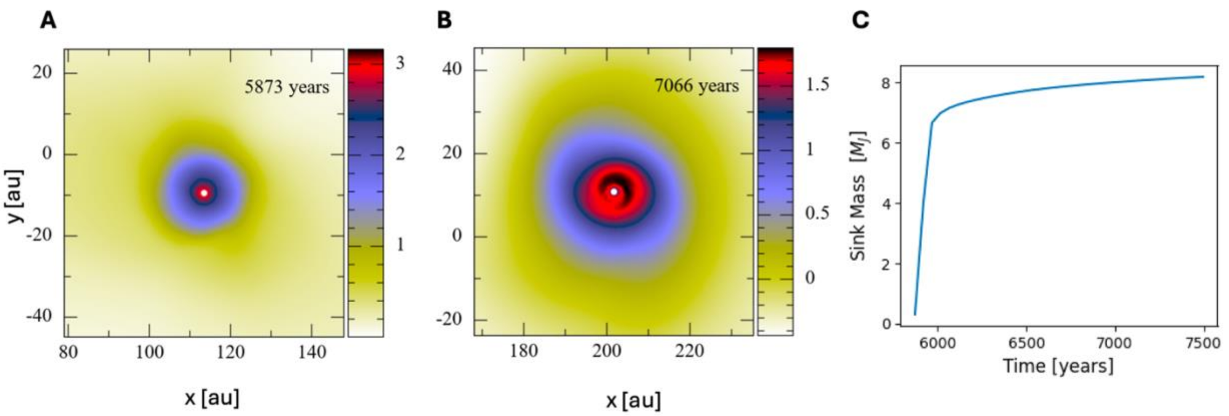

Fig. S4

The evolution of sink A in the rp400v2.5 model of table S1 (see also Fig. 3A). Panels (A) and (B) show the column density map in g/cm^2 in a logarithmic scale; the sink radius is 0.5 au and is not shown to scale in the plots. Panel (C) shows the growth of the sink particle mass where the linear growth stage can be regarded as the spherical collapse phase. Later, sink particles create regions of effective vacuum in the cores of PMOs, leading to exaggerated accretion so that their masses are upper limits of the PMOs' true mass. Increasing the resolution can better resolve the flow around PMOs, resulting in less numerical accretion and lower sink mass (*29*, fig. S5).

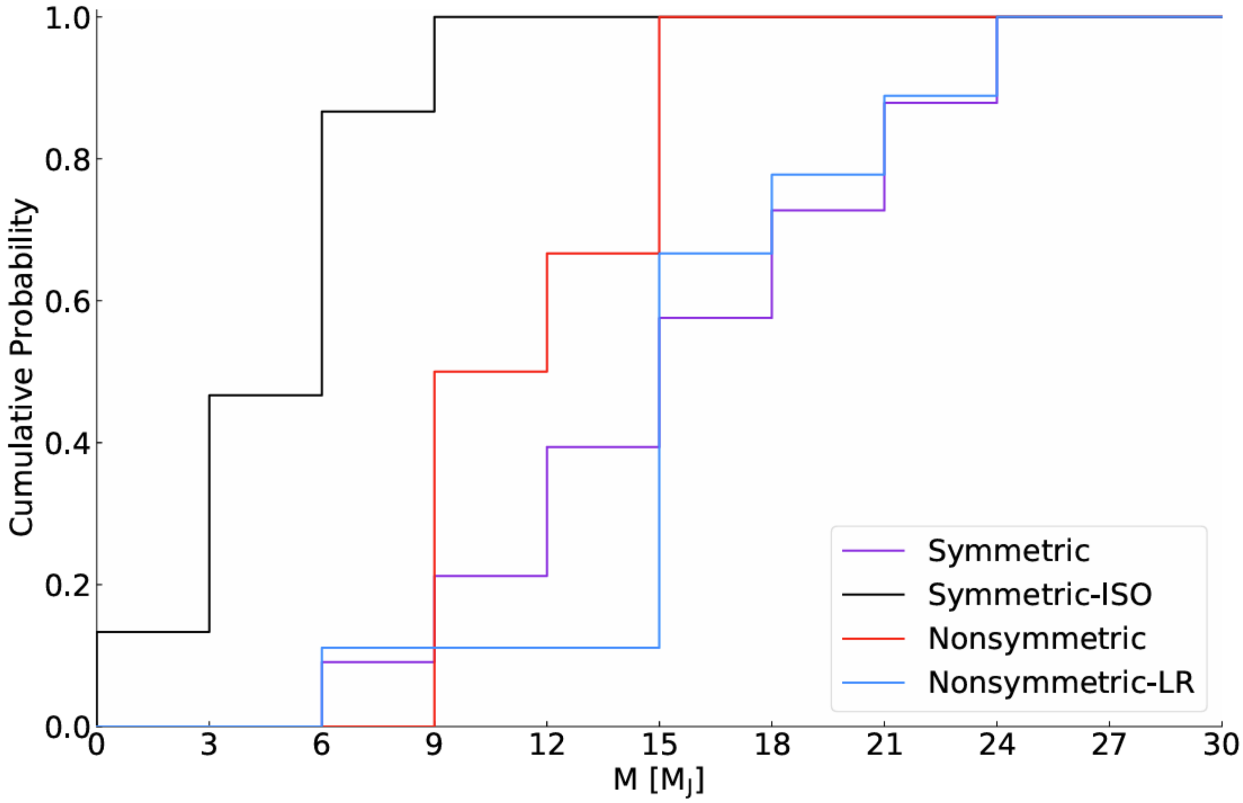

Fig. S5.

Cumulative distribution function of the free-floating objects' (FFOs) mass in symmetric encounters with two identical disks (table S1), symmetric encounter with isothermal disks (table S3), nonsymmetric encounters with two different disks at the fiducial resolution (table S2) and a low resolution (table S6). Nonsymmetric encounters tend to form more PMOs than symmetric encounters, while isothermal simulations form PMOs exclusively. We expect a larger PMO fraction in the barotropic simulations if the resolution is even higher, given the trend in the resolution test (see also discussion in fig. S4). However, the isothermal test cases featuring almost spherical collapse with minor disks (table S3) likely possess the lowest mass PMOs that can form via disk encounters.

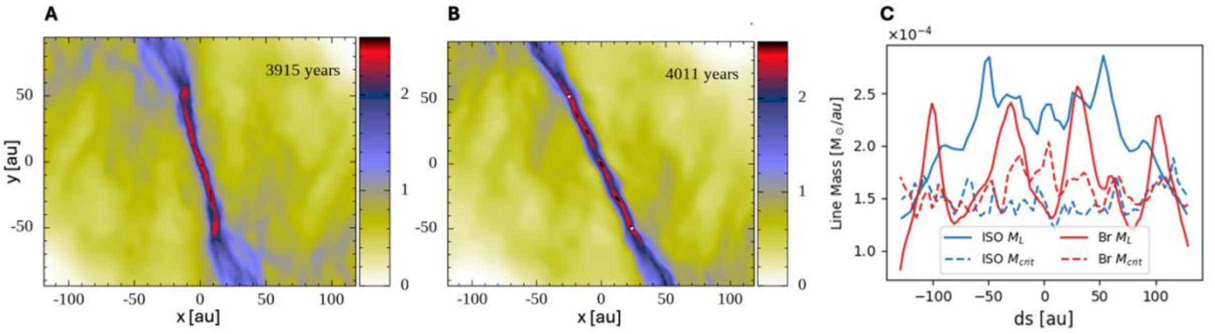

Fig. S6

The filaments in the barotropic (Br) simulation rp400v2.5 (panel A, see table S1) and isothermal (ISO) simulation ir400v2.5 (panel B, see table S3). Panel (C) compares the filaments line mass (solid lines) to the critical line mass for stability (dashed lines). The filament in the isothermal simulation is more conducive to fragmentation and eventually forms 6 closely packed sink particles (see Movie S2).

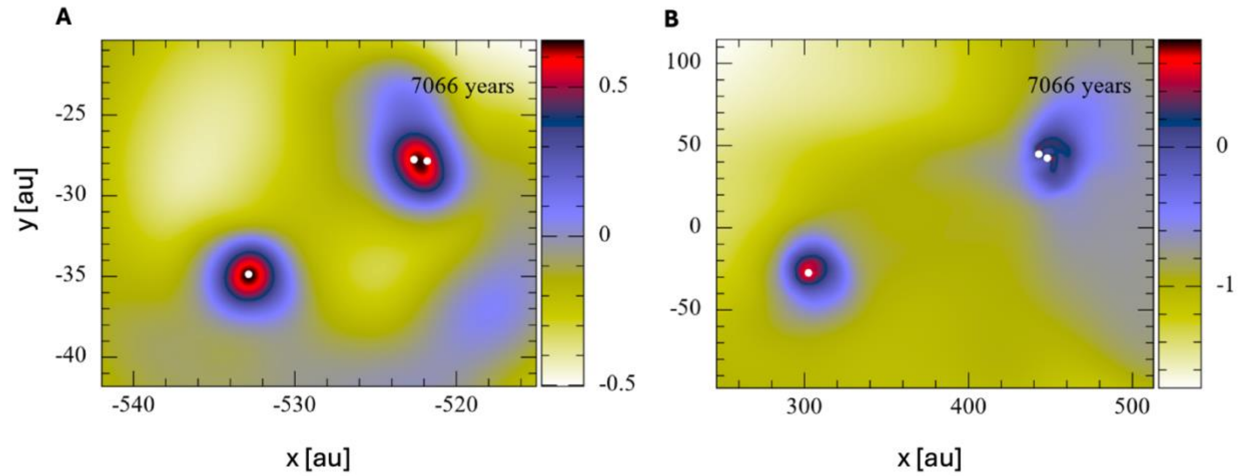

Fig. S7

Two free-floating PMO triples formed in the isothermal simulation ir400v25 of table S3 (see Movie S2). The gas column density is low, and the tight binaries in panels (A) and (B) are not expected to merge, while the loosely bound third PMO in panel (B) may be ionized due to dynamical interactions with other stars (*44*).

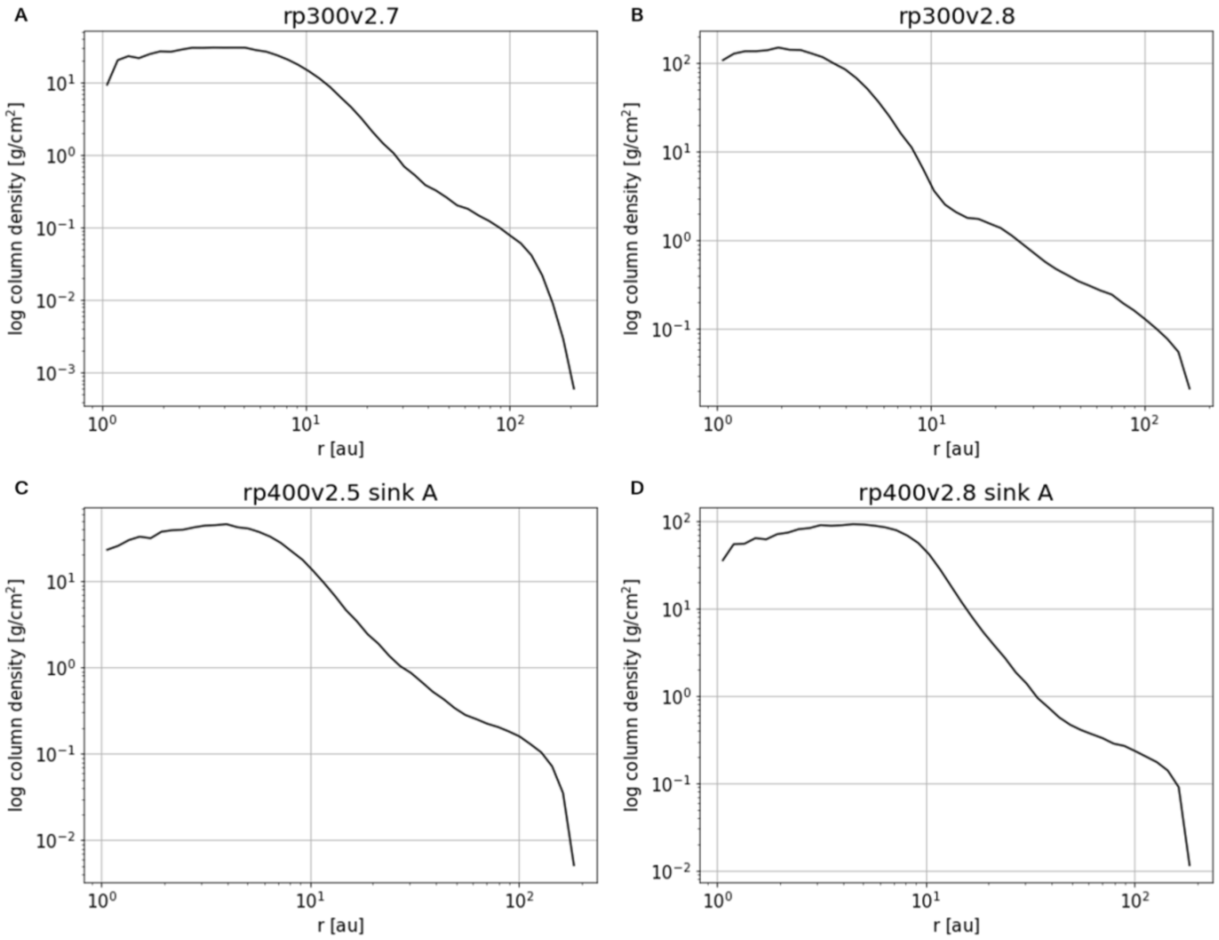

Fig. S8

Panel (A) to (D), surface density profiles of disks around several free-floating single PMOs in table S1. They have similar profiles that feature a flat region followed by fast decay, which is useful for verifying the PMO formation theory via disk encounters.

Table S1.

The fiducial encounter simulations of two 0.3 solar mass stars, each hosting a 0.18 solar mass disk (fig. S1, m_s0.3m_d0.18 model). The simulations are labeled by the encounter orbits' periapsis distance (in au) and velocity at infinity (in km/s). The number of bound companions (BCs) and free-floating objects (FFOs), including binaries are listed; FFOs' mass is given as the sink particle mass (in Jupiter mass, M_J), and all materials within their Hill radii are regarded as their disk. However, it is difficult to define circumsingle disks around multiple FFOs, so we temporarily ignore them. For example, material around single PMOs is substantially ejected during the periapsis crossing of binary PMOs in the rp400v2.65 model (Movie S1).

Case	Number of BCs	Number of FFOs (binaries)	A Sink mass (disk mass) for FFOs	B Sink mass (disk mass) for FFOs
rp200v1	6	0		
rp200v2	3	0		
rp200v3	4	0		
rp200v4	0	0		
rp200v5	0	0		
rp300v1	4	0		
rp300v1.5	5	0		
rp300v2	0	1	26.58 (11.55)	
rp300v2.1	0	1	21.91 (8.66)	
rp300v2.2	2	0		
rp300v2.3	0	1	23.48 (8.47)	
rp300v2.4	0	1	26.46 (15.69)	
rp300v2.5	1	2 (1)	10.3	14.64
rp300v2.6	2	0		
rp300v2.7	0	1	11.33 (2.71)	
rp300v2.8	2	1	7.21 (3.09)	
rp300v2.9	0	1	22.27 (16.65)	
rp300v3	0	1	21.12 (11.7)	
rp300v3.5	0	0		
rp300v4	0	0		
rp300v5	0	0		
rp350v2.2	2	1	19.24 (6.57)	
rp350v2.3	0	1	25.73 (16.29)	
rp350v2.4	0	2 (1)	16.29	16.57

rp350v2.5	0	1	20.17 (14.39)	
rp400v1	3	0		
rp400v1.5	2	1	14.34 (5.03)	
rp400v2	2	1	15.66 (3.56)	
rp400v2.1	0	1	18.79 (12.76)	
rp400v2.2	0	2 (1)	13.54	14.85
rp400v2.3	0	2	17.99 (5.4)	18.03 (5.72)
rp400v2.4	0	1	21.26 (16.64)	
rp400v2.5	0	2	8.02 (3.35)	8.19 (3.38)
rp400v2.6	0	1	17.07 (13.1)	
rp400v2.65	0	2 (1)	12.8	13.24
rp400v2.7	0	0		
rp400v2.8	0	2	11.39 (7.31)	11.6 (7.08)
rp400v2.9	0	1	15.4 (17.58)	
rp400v3	0	1	20.48 (10.47)	
rp400v3.5	0	0		
rp400v4	0	0		
rp500v1	0	0		
rp500v2	0	1	24.03 (13.33)	
rp500v3	0	0		
rp500v4	0	0		
rp500v5	0	0		
rp600v2	0	0		

Table S2.

Encounters between two different stars of 0.3 solar mass and 0.33 solar mass hosting a disk of 0.18 solar mass and 0.2 solar mass (fig. S1), respectively. The nonsymmetric encounters are labeled by the encounter orbits’ periapsis distance and velocity at infinity like table S1.

Case	Number of BCs	Number of FFOs (binaries)	Sink mass (disk mass) for FFOs
nr300v2.1	4	0	
nr300v2.2	1	0	
nr300v2.3	2	0	
nr300v2.4	4	0	
nr300v2.5	1	1	11.02 (3.07)
nr300v2.6	2	1	12.85 (4.22)
nr300v2.7	1	1	12.77 (5.79)
nr300v2.8	2	1	15.94 (4.21)
nr300v2.9	0	1	10.35 (3.88)
nr300v3.0	1	1	9.91 (4.83)
nr400v2.1	1	1	10.86 (2.56)
nr400v2.2	1	0	
nr400v2.3	1	0	
nr400v2.4	1	1	11.67 (5.34)
nr400v2.5	1	0	
nr400v2.6	0	1	16.75 (11.64)
nr400v2.7	1	0	
nr400v2.8	0	1	17.94 (6.72)
nr400v2.9	0	1	10.66 (5.54)
nr400v3.0	0	1	15.19 (7.61)

Table S3.

Test simulations with isothermal EOS involving the m_s0.3m_d0.18-ISO disk in fig. S1, to be compared with table S1. Hierarchical multiple PMOs, including triple (T) and quadruple (Q), are formed due to the interaction of closely packed dense cores (fig. S6, S7).

Case	Number of BCs	Number of FFPs (Multiples)	Sink mass (disk mass) for FFPs	Sink mass (disk mass) for FFPs	Sink mass (disk mass) for FFPs
ir400v2.5	5	6 (2T)	2.99	5.76	6.38
			6.96	9.58	10.58
ir400v2.6	2	7 (1Q)	0.96 (0)	3.2 (0.1)	3.61
			3.67 (0)	4.43	7.14
			7.43		
ir400v3.0	0	2	6.31 (3.47)	6.97 (3.96)	

Table S4.

Detailed information for multiple FFOs. We note that in the fiducial simulations of table S1, FFOs are often slightly above 13 M_J but the sink particle mass is likely an overestimation (fig. S4, S5).

<table>
<tr><th>Case</th><th>m_1 $[M_J]$</th><th>m_2 $[M_J]$</th><th>a_2 [au]</th><th>e_2</th><th>m_3 $[M_J]$</th><th>a_3 [au]</th><th>e_3</th><th>Note</th></tr>
<tr><td>rp300v2.5</td><td>10.3</td><td>14.64</td><td>14.7</td><td>0.65</td><td></td><td></td><td></td><td>Binary</td></tr>
<tr><td>rp350v2.4</td><td>16.29</td><td>16.57</td><td>8.1</td><td>0.27</td><td></td><td></td><td></td><td>Binary</td></tr>
<tr><td>rp400v2.2</td><td>13.54</td><td>14.85</td><td>7.68</td><td>0.27</td><td></td><td></td><td></td><td>Binary</td></tr>
<tr><td>rp400v2.65</td><td>12.8</td><td>13.24</td><td>15.18</td><td>0.52</td><td></td><td></td><td></td><td>Binary</td></tr>
<tr><td rowspan="2">ir400v2.5</td><td>6.38</td><td>10.58</td><td>3.78</td><td>0.53</td><td>6.96</td><td>92.67</td><td>0.84</td><td>Triple</td></tr>
<tr><td>2.99</td><td>5.76</td><td>0.84</td><td>0.03</td><td>9.58</td><td>8.13</td><td>0.66</td><td>Triple</td></tr>
<tr><td rowspan="2">ir400v2.6</td><td>3.61</td><td>7.43</td><td>2.39</td><td>0.55</td><td rowspan="2"></td><td rowspan="2">80.65</td><td rowspan="2">0.71</td><td rowspan="2">Quadruple</td></tr>
<tr><td>4.43</td><td>7.14</td><td>1.84</td><td>0.8</td></tr>
</table>

Table S5.

Test simulations involving two identical disks (m_s0.3m_d0.18 model in fig. S1) with mutual disk inclination of *i* degrees. Encounters with mutual disk inclinations smaller than the disk opening angle, about 5 degrees, can form FFOs.

Case	Number of BCs	Number of FFOs (binaries)	Sink mass (disk mass) for FFOs
rp300v2.5i3	0	1	17.77 (6.77)
rp300v2.5i5	1	1	10.44 (3.64)
rp300v2.5i7	0	0	
rp300v2.5i9	0	0	
rp400v2.5i3	0	1	15.31 (10.64)
rp400v2.5i5	0	0	
rp400v2.5i7	0	0	
rp400v2.5i9	0	0	

Table S6.

Low-resolution simulations involving the two barotropic disk models in fig. S1 but at a mass resolution of 0.0002 Jupiter mass. The results are compared to those of table S2 in fig. S5. In general, the low-resolution simulations form more massive FFOs than the high-resolution simulations.

Case	Number of BCs	Number of FFOs (binaries)	Sink mass (disk mass) for FFOs
nr300v2.1LR	2	0	
nr300v2.2LR	0	1	26 (9.64)
nr300v2.3LR	1	0	
nr300v2.4LR	1	1	17.35 (4.54)
nr300v2.5LR	0	1	17.25 (4.98)
nr300v2.6LR	1	1	15.44 (3.83)
nr300v2.7LR	1	1	15.95 (5.57)
nr300v2.8LR	0	1	22.09 (8.41)
nr300v2.9LR	0	1	17.11 (5.54)
nr300v3.0LR	0	1	7.77 (3.13)
nr300v4.0LR	0	0	
nr400v2.0LR	1	1	20.11 (7.57)
nr400v2.5LR	0	0	
nr400v3.0LR	0	0	

Movie S1.

Animation of Fig. 1. It shows the formation of a free-floating PMO binary in the rp400v2.65 model of table S1.

Movie S2.

Animation of fig. S7. It shows the formation of two free-floating PMO triples in the ir400v2.5 model of table S3.

REFERENCES AND NOTES

1. M. R. Zapatero Osorio, V. J. S. Béjar, E. L. Martín, R. Rebolo, D. B. y Navascués, C. A. L. Bailer-Jones, R. Mundt, Discovery of young, isolated planetary mass objects in the σ orionis star cluster. *Science* **290**, 103–107 (2000).

2. N. Miret-Roig, H. Bouy, S. N. Raymond, M. Tamura, E. Bertin, D. Barrado, J. Olivares, P. A. B. Galli, J.-C. Cuillandre, L. M. Sarro, A. Berihuete, N. Huélamo, A rich population of free-floating planets in the Upper Scorpius young stellar association. *Nat. Astron.* **6**, 89–97 (2022).

3. The Microlensing Observations in Astrophysics (MOA) Collaboration, The Optical Gravitational Lensing Experiment (OGLE) Collaboration, Unbound or distant planetary mass population detected by gravitational microlensing. *Nature* **473**, 349–352 (2011).

4. P. Mróz, A. Udalski, J. Skowron, R. Poleski, S. Kozłowski, M. K. Szymański, I. Soszyński, Ł. Wyrzykowski, P. Pietrukowicz, K. Ulaczyk, D. Skowron, M. Pawlak, No large population of unbound or wide-orbit Jupiter-mass planets. *Nature* **548**, 183–186 (2017).

5. S. G. Pearson, M. J. McCaughrean, Jupiter mass binary objects in the Trapezium cluster. arXiv:2310.01231 [astro-ph.EP] (2023).

6. K. L. Luhman, C. A. de Oliveira, I. Baraffe, G. Chabrier, T. R. Geballe, R. J. Parker, Y. J. Pendleton, P. Tremblin, A JWST survey for planetary mass brown dwarfs in IC 348. *Astron. J.* **167**, 19 (2023).

7. A. B. Langeveld, A. Scholz, K. Mužić, R. Jayawardhana, D. Capela, L. Albert, R. Doyon, L. Flagg, M. de Furio, D. Johnstone, D. Lafrèniere, M. Meyer, The JWST/NIRISS deep spectroscopic survey for young brown dwarfs and free-floating planets. *Astron. J.* **168**, 179 (2024).

8. M. De Furio, M. R. Meyer, T. Greene, K. Hodapp, D. Johnstone, J. Leisenring, M. Rieke, M. Robberto, T. Roellig, G. Cugno, E. Fiorellino, C. Manara, R. Raileanu, S. van Terwisga, Identification of a turnover in the initial mass function of a young stellar cluster down to 0.5 M_J. arXiv:2409.04624 [astro-ph.SR] (2024).

9. A. A. Muench, E. A. Lada, C. J. Lada, J. Alves, The luminosity and mass function of the trapezium cluster: From B stars to the deuterium-burning limit. *Astrophys. J.* **573**, 366–393 (2002).

10. W. M. J. Best, M. C. Liu, T. J. Dupuy, E. A. Magnier, The Young L Dwarf 2MASS J11193254-1137466 is a planetary-mass binary. *Astrophys. J.* **843**, L4 (2017).

11. C. Fontanive, K. N. Allers, B. Pantoja, B. Biller, S. Dubber, Z. Zhang, T. Dupuy, M. C. Liu, L. Albert, A wide planetary-mass companion to a young low-mass brown dwarf in ophiuchus. *Astrophys. J. Lett.* **905**, L14 (2020).

12. E. L. Martín, M. {Ž}erjal, H. Bouy, D. Martin-Gonzalez, S. Mu{ň}oz Torres, D. Barrado, J. Olivares, A. Pérez-Garrido, P. Mas-Buitrago, P. Cruz, E. Solano, M. R. Zapatero Osorio, N. Lodieu, V. J. S. Béjar, J.-Y. Zhang, C. del Burgo, N. Huélamo, R. Laureijs, A. Mora, T. Saifollahi, J.-C. Cuillandre, M. Schirmer, R. Tata, S. Points, N. Phan-Bao, B. Goldman, S. L. Casewell, C. Reylé, R. L. Smart, N. Aghanim, B. Altieri, S. Andreon, N. Auricchio, M. Baldi, A. Balestra, S. Bardelli, A. Basset, R. Bender, D. Bonino, E. Branchini, M. Brescia, J. Brinchmann, S. Camera, V. Capobianco, C. Carbone, J. Carretero, S. Casas, M. Castellano, S. Cavuoti, A. Cimatti, G. Congedo, C. J. Conselice, L. Conversi, Y. Copin, L. Corcione, F. Courbin, H. M. Courtois, M. Cropper, A. Da Silva, H. Degaudenzi, A. M. Di Giorgio, J. Dinis, F. Dubath, X. Dupac, S. Dusini, A. Ealet, M. Farina, S. Farrens, S. Ferriol, P. Fosalba, M. Frailis, E. Franceschi, M. Fumana, S. Galeotta, B. Garilli, W. Gillard, B. Gillis, C. Giocoli, P. Gómez-Alvarez, A. Grazian, F. Grupp, L. Guzzo, S. V. H. Haugan, J. Hoar, H. Hoekstra, W. Holmes, I. Hook, F. Hormuth, A. Hornstrup, D. Hu, P. Hudelot, K. Jahnke, M. Jhabvala, E. Keihänen, S. Kermiche, A. Kiessling, M. Kilbinger, T. Kitching, R. Kohley, B. Kubik, M. Kümmel, M. Kunz, H. Kurki-Suonio, D. Le Mignant, S. Ligori, P. B. Lilje, V. Lindholm, I. Lloro, D. Maino, E. Maiorano, O. Mansutti, O. Marggraf, N. Martinet, F. Marulli, R. Massey, E. Medinaceli, S. Mei, M. Melchior, Y. Mellier, M. Meneghetti, G. Meylan, J. J. Mohr, M. Moresco, L. Moscardini, S.-M. Niemi, C. Padilla, S. Paltani, F. Pasian, K. Pedersen, W. J. Percival, V. Pettorino, S. Pires, G. Polenta, M. Poncet, L. A. Popa, L. Pozzetti, G. D. Racca, F. Raison, R. Rebolo, A. Renzi, J. Rhodes, G. Riccio, H.-W. Rix, E. Romelli, M. Roncarelli, E. Rossetti, R. Saglia, D. Sapone, B. Sartoris, M. Sauvage, R. Scaramella, P. Schneider, A. Secroun, G. Seidel, M. Seiffert, S. Serrano, C. Sirignano, G. Sirri, L. Stanco, P. Tallada-Crespí, A. N. Taylor, H. I. Teplitz, I.

Tereno, R. Toledo-Moreo, A. Tsyganov, I. Tutusaus, L. Valenziano, T. Vassallo, G. Verdoes Kleijn, Y. Wang, J. Weller, O. R. Williams, E. Zucca, C. Baccigalupi, G. Willis, P. Simon, J. Martín-Fleitas, D. Scott, Euclid: Early release observations—A glance at free-floating new-born planets in the sigma Orionis cluster. arXiv:2405.13497 [astro-ph.EP] (2024).

13. S. S. R. Offner, M. Moe, K. M. Kratter, S. I. Sadavoy, E. L. N. Jensen, J. J. Tobin, The origin and evolution of multiple star systems. *ASP Conf. Ser.* **534**, 275 (2023).

14. R. J. Parker, C. Alves de Oliveira, On the origin of planetary-mass objects in NGC 1333. *Mon. Not. R. Astron. Soc.* **525**, 1677–1686 (2023).

15. S. P. Zwart, E. Hochart, The origin and evolution of wide Jupiter mass binary objects in young stellar clusters. *SciPost Astron.* **3**, 1 (2024).

16. M. Fang, J. S. Kim, I. Pascucci, D. Apai, C. F. Manara, A candidate planetary-mass object with a photoevaporating disk in orion. *Astrophys. J. Lett.* **833**, L16 (2016).

17. V. Joergens, M. Bonnefoy, Y. Liu, A. Bayo, S. Wolf, G. Chauvin, P. Rojo, OTS 44: Disk and accretion at the planetary border. *Astron. Astrophys.* **558**, L7 (2013).

18. A. Scholz, K. Muzic, R. Jayawardhana, V. Almendros-Abad, I. Wilson, Disks around Young Planetary-mass Objects: Ultradeep spitzer imaging of NGC 1333. *Astron. J.* **165**,196 (2023).

19. Y. Wang, R. Perna, Z. Zhu, Free-floating binary planets from ejections during close stellar encounters. *Nat. Astron.* **8**, 756–764 (2024).

20. F. Yu, D. Lai, Free-floating planets, survivor planets, captured planets, and binary planets from stellar flybys. *Astrophys. J.* **970**, 97 (2024).

21. A. Vigan, C. Fontanive, M. Meyer, B. Biller, M. Bonavita, M. Feldt, S. Desidera, G.-D. Marleau, A. Emsenhuber, R. Galicher, K. Rice, D. Forgan, C. Mordasini, R. Gratton, H. Le Coroller, A.-L. Maire, F. Cantalloube, G. Chauvin, A. Cheetham, J. Hagelberg, A.-M. Lagrange, M. Langlois, M. Bonnefoy, J.-L. Beuzit, A. Boccaletti, V. D'Orazi, P. Delorme, C. Dominik, T. Henning, M. Janson, E. Lagadec, C. Lazzoni, R. Ligi, F. Menard, D. Mesa, S. Messina, C. Moutou, A. Müller, C. Perrot, M. Samland, H. M. Schmid, T. Schmidt, E. Sissa,

M. Turatto, S. Udry, A. Zurlo, L. Abe, J. Antichi, R. Asensio-Torres, A. Baruffolo, P. Baudoz, J. Baudrand, A. Bazzon, P. Blanchard, A. J. Bohn, S. B. Sevilla, M. Carbillet, M. Carle, E. Cascone, J. Charton, R. Claudi, A. Costille, V. De Caprio, A. Delboulbé, K. Dohlen, N. Engler, D. Fantinel, P. Feautrier, T. Fusco, P. Gigan, J. H. Girard, E. Giro, D. Gisler, L. Gluck, C. Gry, N. Hubin, E. Hugot, M. Jaquet, M. Kasper, D. Le Mignant, M. Llored, F. Madec, Y. Magnard, P. Martinez, D. Maurel, O. Möller-Nilsson, D. Mouillet, T. Moulin, A. Origné, A. Pavlov, D. Perret, C. Petit, J. Pragt, P. Puget, P. Rabou, J. Ramos, E. L. Rickman, F. Rigal, S. Rochat, R. Roelfsema, G. Rousset, A. Roux, B. Salasnich, J.-F. Sauvage, A. Sevin, C. Soenke, E. Stadler, M. Suarez, Z. Wahhaj, L. Weber, F. Wildi, The SPHERE infrared survey for exoplanets (SHINE). III. The demographics of young giant exoplanets below 300 au with SPHERE. *Astron. Astrophys.* **651**, A72 (2021).

22. P. André, J. Di Francesco, D. Ward-Thompson, S.-I. Inutsuka, R. E. Pudritz, J. E. Pineda, "From filamentary networks to dense cores in molecular clouds: Toward a new paradigm for star formation" in *Protostars and Planets VI*, H. Beuther, R. Klessen, C. Dullemond, Th. Henning, Eds. (University of Arizona Press, 2014), pp. 27–52.

23. S. Inutsuka, S. M. Miyama, A production mechanism for clusters of dense cores. *Astrophys. J.* **480**, 681–693 (1997).

24. P. Palmeirim, P. André, J. Kirk, D. Ward-Thompson, D. Arzoumanian, V. Könyves, P. Didelon, N. Schneider, M. Benedettini, S. Bontemps, J. Di Francesco, D. Elia, M. Griffin, M. Hennemann, T. Hill, P. G. Martin, A. Men'shchikov, S. Molinari, F. Motte, Q. N. Luong, D. Nutter, N. Peretto, S. Pezzuto, A. Roy, K. L. J. Rygl, L. Spinoglio, G. L. White, *Herschel* view of the Taurus B211/3 filament and striations: Evidence of filamentary growth? *Astron. Astrophys.* **550**, A38 (2013).

25. K. Kratter, G. Lodato, Gravitational instabilities in circumstellar disks. *Annu. Rev. Astron. Astrophys.* **54**, 271–311 (2016).

26. W. Xu, Testing a new model of embedded protostellar disks against observations: The majority of orion class 0/I disks are likely warm, massive, and gravitationally unstable. *Astrophys. J.* **934**, 156 (2022).

27. L. Mayer, T. Quinn, J. Wadsley, J. Stadel, Formation of giant planets by fragmentation of protoplanetary disks. *Science* **298**, 1756–1759 (2002).

28. H. Deng, L. Mayer, R. Helled, Formation of intermediate-mass planets via magnetically controlled disk fragmentation. *Nat. Astron.* **5**, 440–444 (2021).

29. I. Thies, P. Kroupa, S. P. Goodwin, D. Stamatellos, A. P. Whitworth, Tidally induced brown dwarf and planet formation in circumstellar disks. *Astrophys. J.* **717**, 577–585 (2010).

30. S. J. Watkins, A. S. Bhattal, H. M. J. Boffin, N. Francis, A. P. Whitworth, Numerical simulations of protostellar encounters—II. Coplanar disc-disc encounters. *Mon. Not. R. Astron. Soc.* **300**, 1205–1213 (1998).

31. D. N. C. Lin, G. Laughlin, P. Bodenheimer, M. Rozyczka, The formation of substellar objects induced by the collision of protostellar disks. *Science* **281**, 2025–2027 (1998).

32. S. Shen, J. Wadsley, T. Hayfield, N. Ellens, A numerical study of brown dwarf formation via encounters of protostellar discs. *Mon. Not. R. Astron. Soc.* **401**, 727–742 (2010).

33. A. F. Nelson, Numerical requirements for simulations of self-gravitating and non-self-gravitating discs. *Mon. Not. R. Astron. Soc.* **373**, 1039–1073 (2006).

34. I. Backus, T. Quinn, Fragmentation of protoplanetary discs around M-dwarfs. *Mon. Not. R. Astron. Soc.* **463**, 2480–2493 (2016).

35. P. F. Hopkins, A new class of accurate, mesh-free hydrodynamic simulation methods. *Mon. Not. R. Astron. Soc.* **450**, 53–110 (2015).

36. A. Toomre, J. Toomre, Galactic bridges and tails. *Astrophys. J.* **178**, 623–666 (1972).

37. E. D'Onghia, M. Vogelsberger, C.-A. Faucher-Giguere, L. Hernquist, Quasi-resonant theory of tidal interactions. *Astrophys. J.* **725**, 353–368 (2010).

38. S. M. Vicente, J. Alves, Size distribution of circumstellar disks in the Trapezium cluster. *Astron. Astrophys.* **441**, 195–205 (2005).

39. M. J. McCaughrean, J. R. Stauffer, High resolution near-infrared imaging of the trapezium: A stellar census. *Astron. J.* **108**, 1382 (1994).

40. L. A. Hillenbrand, L. W. Hartmann, A preliminary study of the orion nebula cluster structure and dynamics. *Astrophys. J.* **492**, 540–553 (1998).

41. M. A. Kuhn, L. A. Hillenbrand, A. Sills, E. D. Feigelson, K. V. Getman, Kinematics in young star clusters and associations with Gaia DR2. *Astrophys. J.* **870**, 32 (2019).

42. M. R. Bate, I. A. Bonnell, N. M. Price, Modelling accretion in protobinary systems. *Mon. Not. R. Astron. Soc.* **277**, 362–376 (1995).

43. M. Y. Grudić, D. Guszejnov, P. F. Hopkins, S. S. R. Offner, C.-A. Faucher-Giguère, STARFORGE: Towards a comprehensive numerical model of star cluster formation and feedback. *Mon. Not. R. Astron. Soc.* **506**, 2199–2231 (2021).

44. Y. Huang, W. Zhu, E. Kokubo, Dynamics of binary planets within star clusters. *Astrophys. J. Lett.* **975**, L38 (2024).

45. J. Huang, E. A. Bergin, K. I. Öberg, S. M. Andrews, R. Teague, C. J. Law, P. Kalas, Y. Aikawa, J. Bae, J. B. Bergner, A. S. Booth, A. D. Bosman, J. K. Calahan, G. Cataldi, L. I. Cleeves, I. Czekala, J. D. Ilee, R. Le Gal, V. V. Guzmán, F. Long, R. A. Loomis, F. Ménard, H. Nomura, C. Qi, K. R. Schwarz, T. Tsukagoshi, M. L. R. van't Hoff, C. Walsh, D. J. Wilner, Y. Yamato,K. Zhang, Molecules with ALMA at planet-forming scales (MAPS). XIX. Spiral arms, a tail, and diffuse structures traced by CO around the GM Aur disk. *Astrophys. J. Suppl. Ser.* **257**, 19 (2021).

46. O. De Marco, C. R. O'Dell, P. Gelfond, R. H. Rubin, S. C. O. Glover, Cloud fragmentation and proplyd-like features in H II regions imaged by the hubble space telescope. *Astron. J.* **131**, 2580–2600 (2006).

47. M. R. Bate, On the diversity and statistical properties of protostellar discs. *Mon. Not. R. Astron. Soc.* **475**, 5618–5658 (2018).

48. N. Miret-Roig, P. A. B. Galli, J. Olivares, H. Bouy, J. Alves, D. Barrado, The star formation history of Upper Scorpius and Ophiuchus. A 7D picture: Positions, kinematics, and dynamical traceback ages. *Astron. Astrophys.* **667**, A163 (2022).

49. M. Cottaar, K. R. Covey, J. B. Foster, M. R. Meyer, J. C. Tan, D. L. Nidever, S. D. Chojnowski, N. da Rio, K. M. Flaherty, P. M. Frinchaboy, S. Majewski, M. F. Skrutskie, J. C. Wilson, G. Zasowski, IN-SYNC. III. The dynamical state of IC 348—A super-virial velocity dispersion and a puzzling sign of convergence. *Astrophys. J.* **807**, 27 (2015).

50. J. B. Foster, M. Cottaar, K. R. Covey, H. G. Arce, M. R. Meyer, D. L. Nidever, K. G. Stassun, J. C. Tan, S. D. Chojnowski, N. da Rio, K. M. Flaherty, L. Rebull, P. M. Frinchaboy, S. R. Majewski, M. Skrutskie, J. C. Wilson, G. Zasowski, IN-SYNC. II. Virial stars from subvirial cores—The velocity dispersion of embedded pre-main-sequence Stars in NGC 1333. *Astrophys. J.* **799**, 136 (2015).

51. V. Springel, The cosmological simulation code GADGET-2. *Mon. Not. R. Astron. Soc.* **364**, 1105–1134 (2005).

52. D. J. Price, J. J. Monaghan, An energy-conserving formalism for adaptive gravitational force softening in smoothed particle hydrodynamics and N-body codes. *Mon. Not. R. Astron. Soc.* **374**, 1347–1358 (2007).

53. H. Deng, L. Mayer, F. Meru, Convergence of the critical cooling rate for protoplanetary disk fragmentation achieved: The key role of numerical dissipation of angular momentum. *Astrophys. J.* **847**, 43 (2017).

54. C. Federrath, M. Schrön, R. Banerjee, R. S. Klessen, Modeling jet and outflow feedback during star cluster formation. *Astrophys. J.* **790**, 128 (2014).

55. H. Masunaga, S. Inutsuka, A radiation hydrodynamic model for protostellar collapse. II. The second collapse and the birth of a protostar. *Astrophys. J.* **531**, 350–365 (2000).

56. D. Stamatellos, A. P. Whitworth, The role of thermodynamics in disc fragmentation. *Mon. Not. R. Astron. Soc.* **400**, 1563–1573 (2009).

57. J. Speedie, R. Dong, C. Hall, C. Longarini, B. Veronesi, T. Paneque-Carreño, G. Lodato, Y.-W. Tang, R. Teague, J. Hashimoto, Gravitational instability in a planet-forming disk. *Nature* **633**, 58–62 (2024).

58. J. P. Williams, C. Painter, A. R. Anderson, A. Ribas, Dust drift timescales in protoplanetary disks at the cusp of gravitational instability. *Astrophys. J.* **976**, 50 (2024).

59. D. Lynden-Bell, J. E. Pringle, The evolution of viscous discs and the origin of the nebular variables. *Mon. Not. R. Astron. Soc.* **168**, 603–637 (1974).

60. S. M. Andrews, D. J. Wilner, A. M. Hughes, C. Qi, C. P. Dullemond, Protoplanetary disk structures in ophiuchus. *Astrophys. J.* **700**, 1502–1523 (2009).

61. A. Toomre, On the gravitational stability of a disk of stars. *Astrophys. J.* **139**, 1217–1238 (1964).

62. A. J. Winter, T. J. Haworth, The external photoevaporation of planet-forming discs. *Eur. Phys. J. Plus* **137**, 1132 (2022).

63. D. J. Muñoz, K. Kratter, M. Vogelsberger, L. Hernquist, V. Springel, Stellar orbit evolution in close circumstellar disc encounters. *Mon. Not. R. Astron. Soc.* **446**, 2010–2029 (2015).

64. Y. Misugi, S. Inutsuka, D. Arzoumanian, Y. Tsukamoto, Evolution of the angular momentum of molecular cloud cores in magnetized molecular filaments. *Astrophys. J.* **963**, 106 (2024).

65. C. F. Manara, M. Tazzari, F. Long, G. J. Herczeg, G. Lodato, A. A. Rota, P. Cazzoletti, G. van der Plas, P. Pinilla, G. Dipierro, S. Edwards, D. Harsono, D. Johnstone, Y. Liu, F. Menard, B. Nisini, E. Ragusa, Y. Boehler, S. Cabrit, Observational constraints on dust disk sizes in tidally truncated protoplanetary disks in multiple systems in the Taurus region. *Astron. Astrophys.* **628**, A95 (2019).

66. E. L. N. Jensen, R. D. Mathieu, A. X. Donar, A. Dullighan, Testing protoplanetary disk alignment in young binaries. *Astrophys. J.* **600**, 789–803 (2004).

67. M. R. Bate, Stellar, brown dwarf and multiple star properties from a radiation hydrodynamical simulation of star cluster formation. *Mon. Not. R. Astron. Soc.* **419**, 3115–3146 (2012).

68. E. I. Vorobyov, S. Basu, The burst mode of accretion and disk fragmentation in the early embedded stages of star formation. *Astrophys. J.* **719**, 1896–1911 (2010).

69. H.-W. Yen, P.-G. Gu, N. Hirano, P. M. Koch, C.-F. Lee, H. B. Liu, S. Takakuwa, HL Tau disk in HCO^+ (3–2) and (1–0) with ALMA: Gas density, temperature, gap, and one-arm spiral. *Astrophys. J.* **880**, 69 (2019).

70. A. S. Booth, J. D. Ilee, $^{13}C^{17}O$ suggests gravitational instability in the HL Tau disc. *Mon. Not. R. Astron. Soc. Lett.* **493**, L108–L113 (2020).

71. B. Veronesi, T. Paneque-Carreño, G. Lodato, L. Testi, L. M. Pérez, G. Bertin, C. Hall, A dynamical measurement of the disk mass in Elias 2–27. *Astrophys. J. Lett.* **914**, L27 (2021).
